# Novel IgG-Degrading Enzymes of the IgdE Protease Family Link Substrate Specificity to Host Tropism of *Streptococcus* Species

**DOI:** 10.1371/journal.pone.0164809

**Published:** 2016-10-17

**Authors:** Christian Spoerry, Pontus Hessle, Melanie J. Lewis, Lois Paton, Jenny M. Woof, Ulrich von Pawel-Rammingen

**Affiliations:** 1 Department of Molecular Biology and Umeå Centre for Microbial Research, Umeå University, Umeå, Sweden; 2 Cell Signalling and Immunology, School of Life Sciences, University of Dundee, Wellcome Trust Building, Dundee, United Kingdom; Uniwersytet Gdanski, POLAND

## Abstract

Recently we have discovered an IgG degrading enzyme of the endemic pig pathogen *S*. *suis* designated IgdE that is highly specific for porcine IgG. This protease is the founding member of a novel cysteine protease family assigned C113 in the MEROPS peptidase database. Bioinformatical analyses revealed putative members of the IgdE protease family in eight other *Streptococcus* species. The genes of the putative IgdE family proteases of *S*. *agalactiae*, *S*. *porcinus*, *S*. *pseudoporcinus* and *S*. *equi* subsp. *zooepidemicus* were cloned for production of recombinant protein into expression vectors. Recombinant proteins of all four IgdE family proteases were proteolytically active against IgG of the respective *Streptococcus* species hosts, but not against IgG from other tested species or other classes of immunoglobulins, thereby linking the substrate specificity to the known host tropism. The novel IgdE family proteases of *S*. *agalactiae*, *S*. *pseudoporcinus* and *S*. *equi* showed IgG subtype specificity, *i*.*e*. IgdE from *S*. *agalactiae* and *S*. *pseudoporcinus* cleaved human IgG1, while IgdE from *S*. *equi* was subtype specific for equine IgG7. Porcine IgG subtype specificities of the IgdE family proteases of *S*. *porcinus* and *S*. *pseudoporcinus* remain to be determined. Cleavage of porcine IgG by IgdE of *S*. *pseudoporcinus* is suggested to be an evolutionary remaining activity reflecting ancestry of the human pathogen to the porcine pathogen *S*. *porcinus*. The IgG subtype specificity of bacterial proteases indicates the special importance of these IgG subtypes in counteracting infection or colonization and opportunistic streptococci neutralize such antibodies through expression of IgdE family proteases as putative immune evasion factors. We suggest that IgdE family proteases might be valid vaccine targets against streptococci of both human and veterinary medical concerns and could also be of therapeutic as well as biotechnological use.

## Introduction

Gram-positive bacteria of the genus *Streptococcus* are highly intertwined with humans and animals as commensal, opportunistic and pathogenic bacteria. Often streptococci show pronounced host tropism, but these bacteria can also cause zoonotic or anthroponotic infections in more uncommon hosts [1].

Starting with the observation of a Immunoglobulin (Ig) G degrading activity in culture supernatants of the important endemic pig pathogen *Streptococcus* (*S*.) *suis*, we identified recently a novel IgG degrading enzyme designated IgdE [2]. Through inhibitor screening, *in silico* modeling and mutational studies of the potential catalytic triad residues, IgdE was assigned to be a cysteine protease. This protease does not have homology to any protease previously described and is thereby the founding member of a novel cysteine protease family designated C113 within the CA clan in the MEROPS peptidase database (https://merops.sanger.ac.uk/) [3]. IgdE of *S*. *suis* is highly specific for porcine IgG and no other substrate has been identified. Immune evasion from Ig mediated immune defense seems to be of special importance for bacteria of the *Streptococcus* genus. Several Ig degrading enzymes of streptococci have been identified, such as the IgG specific proteases, IdeS of *S*. *pyogenes* [4], IdeZ of *S*. *equi* subsp. *zooepidemicus* and IdeE of *S*. *equi* subsp. *equi* [5]. In addition IgA-specific proteases of *S*. *pneumoniae*, *S*. *oralis*, *S*. *sanguis and S*.*mitis* have been described [6, 7] and recently, we described an IgM specific protease in *S*. *suis*, designated IdeSsuis [8]. Most of these proteases cleave the heavy chain of Ig molecules in the hinge region, thereby impairing all effector functions of Ig except neutralization.

IgG is the major antibody in serum of most higher organisms [9, 10]. Human IgG is categorized into four different subclasses; IgG1, IgG2, IgG3 and IgG4, with abundance in the same order [11]. These subclasses have subtle variations in structure resulting in different effector mechanisms. IgG1 and IgG3 are the prevalent Ig classes in humans able to cross the placental barrier to protect the fetus and newborn by passive immunization [12]. Every IgG subclass has an individual FcγR-binding profile with IgG1 and IgG3 binding efficiently to most FcγR, while IgG2 and IgG4 have a reduced affinity for some FcγR [13]. IgG1 and IgG3 activate the classical complement pathway efficiently compared to IgG2 and IgG4 [14–16]. Consequently IgG1 and IgG3 are more involved in complement activation, phagocyte binding, sensitization of mast cells and sensitization of natural killer cells for killing, while all four subclasses are equally involved in neutralization, opsonization and extravascular diffusion [17].

Porcine IgG is divided into six subclasses [18]. These subclasses are considered to be biochemically inseparable. Thus the different IgG subtypes and their proposed properties are only predicted by sequence analyses [19]. Equine IgG is divided into seven subclasses [20]. Experimental studies with recombinant equine IgG subclasses revealed that IgG1, IgG3, IgG4 and IgG7 are the most potent activators of the classical complement pathway via C1q binding and elicit also a strong respiratory burst from equine peripheral blood leukocytes [21].

In this study, we employed rigorous homology searches to identify several homologues of IgdE of *S*. *suis* in other *Streptococcus* species as putative IgdE family proteases and compared them by phylogenetic analysis. The *igdE* genes of *S*. *agalactiae*, *S*. *porcinus*, *S*. *pseudoporcinus* and *S*. *equi* subsp. *zooepidemicus* were cloned for expression and purification of recombinant protein followed by screening for potential substrates of these putative proteases.

*S*. *agalactiae*, also known as Group B *Streptococcus*, is commonly found as a commensal in cattle and humans [22, 23], but is also able to cause mastitis in cows [24] and genitourinary infections, neonatal sepsis, CNS infections and endocarditis in humans [25].

*S*. *porcinus* is a bacterium most commonly found in the respiratory tract of pigs [26] and has been associated with lymphadenitis [27] and still birth [28]. *S*. *pseudoporcinus* has recently been distinguished from *S*. *porcinus* as a separate species [29] and has been shown to be an emerging and common organism colonizing the genitourinary tract of women [30].

*S*. *equi* subsp. *zooepidemicus*, a commensal and opportunistic pathogen of horses as well as other mammals, can cause severe zoonotic infections in humans, such as sepsis, meningitis and endocarditis [31]. In horses *S*. *equi* subsp. *zooepidemicus* can cause several different pathologies, including respiratory tract infections, uteritis and wound infections. The cause of the highly contagious upper respiratory tract disease strangles, *S*. *equi* subsp. *equi*, is believed to be a clonal descendent of an ancestral strain of *S*. *equi* subsp. *zooepidemicus* [32, 33].

All putative IgdE family proteases tested in this study showed enzymatic activity and substrate specificity for IgG of specific hosts. IgdE of *S*. *agalactiae* was specific for human IgG1. IgdE of *S*. *porcinus* was specific for porcine IgG, while IgdE of *S*. *equi* subsp. *zooepidemicus* was specific for equine IgG7. IgdE of *S*. *pseudoporcinus* degraded both human IgG1 as well as porcine IgG thereby being the only identified IgdE family protease with multiple substrates. The substrate specificities, in regard of host species IgG, of these novel members of the IgdE protease family correlate well with the known host tropism of the respective *Streptococcus* species. The IgG subclass specificities of these proteases might implicate special importance of these specific IgG subtypes in immune defense against these *Streptococcus* species during certain stages of infection which the bacteria might counteract through expression of IgG subtype specific proteases.

The IgdE proteases of *S*. *agalactiae*, *S*. *porcinus*, *S*. *pseudoporcinus* and *S*. *equi* subsp. *zooepidemicus* are designated IgdE_agalactiae_, IgdE_porcinus_, IgdE_pseudoporcinus_ and IgdE_equi_ in this study.

## Materials and Methods

### Computational identification of novel IgdE protease family members within Streptococci

Coding sequences of all available *Streptococcus* genomes were downloaded from NCBI (ftp://ftp.ncbi.nlm.nih.gov/genomes/Bacteria/ on Aug-21-2015) and from PATRIC (ftp://ftp.patricbrc.org/patric2/ on Aug-25-2015). As a reference sequence for an IgdE protease the RefSeq sequence WP_014636499.1 of *S*. *suis* was used. The N-terminal signal peptide and the C-terminal region only present in sequences from *S*. *sui*s were removed, leaving amino acids 38–520, hereafter called IgdE_domain.

The IgdE_domain was used as query sequence in blastp searches (E-value cutoff 1 to keep all possible proteases) against the NCBI sequences as well as the PATRIC sequences. Sequences not containing the catalytic cysteine were removed from further consideration. Many of the sequences found are annotated as S-layer proteins or as containing an S-layer homology domain W. These are often present in two or more copies in the same genome, and have an SxC or GxC motif in the catalytic site instead of the AxC motif found in aa 300–302 of the original IgdE sequence of *S*. *suis*. In order to distinguish these sequences, which are not members of the IgdE protease family, all sequences lacking the AxC motif were also removed.

The obtained hits were in turn used as query sequences against the same databases, using the same parameters. From the list of matched sequences those that in the second round had a match overlapping with the region matched in the first round, when the IgdE_domain was used as query, were chosen. Sequences matching to the Transglutcore model with an E-value of at most 1e-6 or sequences not containing the catalytic AxC motif were excluded. The remaining sequences were trimmed at both ends to contain only the parts matching the IgdE_domain sequence. In cases where this resulted in identical sequences only one copy was kept.

### Phylogenetic analysis

Clustal Omega version 1.2.1 (http://www.ebi.ac.uk/Tools/msa/clustalo/) [34] was used to generate a multiple sequence alignment of the sequences obtained above using default settings. To determine the best fitting amino acid substitution model we used ProtTest version 3.4 [35]. A Jones-Taylor-Thornton (JTT) model with a gamma distribution, a proportion of invariable sites, and observed amino acid frequencies was the best model, and therefore used to construct a maximum likelihood (ML) tree with PhyML version 20131022 [36]. To assess the significance of phylogenetic grouping a bootstrap analysis with 100 repetitions was performed. The tree was rooted using an out-group consisting of two non-streptococcal protein sequences homologous to the IgdE_domain (WP_029500965.1; WP_016310821.1). These sequences were trimmed at both ends to contain only the parts matching the IgdE_domain sequence. Encoded proteins lack described functions. The phylogenetic tree was visualized with iTOL (http://itol.embl.de/) [37].

### Bacterial strains and growth conditions

*Escherichia coli* strains were cultured in Lysogeny Broth (LB) or Lysogeny Agar (LA) under aerobic conditions at 30°C or 37°C. When appropriate, 50 μg/ml kanamycin or 25 μg/ml chloramphenicol was added.

### Cloning of IgdE homologues for recombinant expression

Genes of the IgdE homologues lacking the signal peptide were amplified from chromosomal DNA of *S*. *porcinus* strain DSM20725 (kindly received from Christoph G. Bauns, College of Veterinary Medicine, University Leipzig, Leipzig, Germany), *S*. *pseudoporcinus* strain LQ940-04T (ATCC), *S*. *agalactiae* strain CCUG420 (kindly received from Åsa Gylfe, Department of Clinical Microbiology, Umeå University, Umeå, Sweden) and *S*. *equi* subsp. *zooepidemicus* strain 203 (kindly received from National Veterinary Institute, Uppsala, Sweden) as templates using primer pairs designated in Table 1. PCR fragments were cloned into pET_ZZ_1a after digestion with restriction enzymes (all Thermo Scientific) denoted in primer names. The cloned plasmids were verified by sequencing and transformed into *E*. *coli* BL21 (DE3) p*LysS* for recombinant expression of the proteins.

**Table 1:** Used primers for cloning of *igdE* genes.

	Locus tag	aa	primers	
***igdE***_***porcinus***_	STRPO_RS07810	34–527	IgdE_porcinus_-frw_NcoI	GTACCCATGGCTGTTCTTGCGAGAGAAAATAG
			IgdE_porcinus_-rev_Acc65I	GTACGGTACCTTAGTTACCTGCATTCTTTGTTTC
***igdE***_***pseudoporcinus***_	STRPS_RS03610	38–535	IgdE_pseudoporcinus_-frw_Eco31I	GTACGGTCTCCCATGAGAGAAAATGAAAACGTAAGACAATTAC
			IgdE_pseudoporcinus_-rev_Acc65I	GTACGGTACCTTACTGTGCATGCTTTGTTGTTG
***igdE***_***agalactiae***_	MSA_19930	37–623	IgdE_agalactiae_-frw_BspHI	GTACTCATGAATCAAAATAATATTCAAGAAACT
			IgdE_agalactiae_-rev_Acc65I	GTACGGTACCTAATTCGTGTTCGTTTCTC
***igdE***_***equi***_	M837_01916	1-517(no signal peptide)	IgdE_equi_-frw_NcoI	GTACCCATGGAAGCATGGAAGCATG
			IgdE_equi_-rev_Acc65I	GTACGGTACCTTATTGATTAGCGCTTTCACATTG

### Expression and purification of recombinant IgdE homologues

*E*. *coli* BL21(DE3) p*LysS* isolates carrying pET_ZZ_1a *igdE*_*porcinus*_, *igdE*_*pseudoporcinus*_, *igdE*_*agalactiae*_ or *igdE*_*equi*_ were grown to OD600 0.5 at 30°C. Protein expression was induced with 0.5 mM IPTG for 5h at 30°C. Cells were lysed for crude soluble extracts by BugBuster HT Protein Extraction Reagent (Novagen) according to manufacture protocol or lysed by sonication in 20 mM sodium phosphate, 0.5M NaCl, 40 mM imidazole, pH 7.4 prior to further purification. The His-ZZ-tagged proteins were purified on HisTrap FF (GE Healthcare) using standard protocols. The tag was removed by enzymatic cleavage by Tev-protease for 20h at 4°C followed by a second round of purification on HisTrap FF (GE Healthcare). The flow through, containing untagged recombinant protein, was collected and buffer exchanged against PBS. Protein concentrations were determined by Nanodrop A280 measurements at appropriate dilutions. In case of IgdE_equi_ no great overexpression was achieved and no purification attempt was conducted.

### Screening for Ig-degrading activities of recombinant IgdE homologues

If not stated otherwise, all reactions were carried out at 37°C for 16h in PBS. 20 μg/ml purified recombinant proteins or 5% crude soluble extracts of *E*. *coli* expressing the *igdE* constructs were incubated with 0.5 mg/ml porcine, human, bovine, horse, goat, and mouse IgG (all Sigma), 0.25 mg/ml human IgG1 kappa, IgG2 kappa, IgG3 kappa, IgG4 kappa, IgA and IgM (all Sigma), 0.09 mg/ml purified recombinant horse IgG1, IgG2, IgG3, IgG4, IgG5, IgG6 and IgG7 expressed in Chinese hamster ovary cells or FreeStyle 293-F cells (according to [21]) and 1% human plasma, porcine plasma (kindly received from Christoph G. Baums, College of Veterinary Medicine, University Leipzig, Leipzig, Germany) and equine serum (Sigma). Reaction samples were analyzed using SDS-PAGE or Western Blot analyses. Experiments were repeated at least two times and representative analyses are shown.

### SDS-PAGE and Western Blot analysis

Samples for SDS-PAGE were prepared with reducing sample buffer and heated to 95°C for 5 min. 12% SDS-PAGE was either stained with Coomassie blue (Sigma), Coomassie Fluor^™^ Orange Protein Gel Stain (Invitrogen) or blotted to Hybond-P PVDF membrane (GE Healthcare) for Western Blot analyses. Membranes were blocked with 5% dry milk powder in 0.1% PBS-Tween, followed by incubation with horse-radish peroxidase conjugated primary antibodies or unconjugated primary antibody (according to Table 2). Membranes were thoroughly washed with 0.1% PBS-Tween and development with Amersham ECL Select Western blotting detection reagent (GE Healthcare) according to manufacturer’s instruction and chemiluminescent signal was captured by LAS4000 imaging system (Fujifilm). Prestained protein ladders were pictured with the same system by standard epi-white illumination.

**Table 2:** Used antibodies and dilutions for Western Blot analysis.

Name	Manufacturer	Dilution
Goat anti-pig IgG-HRP	Thermo Scientific (PA1-28685)	1:30′000
Goat anti-pig IgM-HRP	Thermo Scientific (PA1-84622)	1:30′000
Goat anti-pig IgA-HRP	Thermo Scientific (PA1-84625)	1:15′000
Rabbit anti-horse IgG-HRP	Abcam (ab6921)	1:30’000
Goat anti-horse IgM-HRP	Abcam (ab112879)	1:15’000
Rabbit anti-horse IgA-HRP	Abcam (ab112871)	1:15’000

### N-terminal Edman sequencing

IgG degradation reactions were separated by SDS-PAGE as previously explained and transferred to PVDF blotting membrane (GE Health Care) by semi-dry blotting with blotting buffer consisting of 50 mM Sodium borate and 20% Methanol. The membrane was stained with Ponceau S 0.5% (Sigma) 1% acetic acid in MQ water and destained with MQ water. The membrane was quickly dried, where after the degradation product was neatly cut out. N-terminal Edman sequencing of the degradation product was performed by Proteome Factory (Berlin, Germany). BLAST homology searches were used to identify the position of the obtained sequence in the IgG molecule.

## Results

### Putative IgdE family proteases are spread among several *Streptococcus* species

To identify novel IgdE family proteases, streptococcal genomes were searched for sequences encoding homologues of the IgdE_domain. The IgdE_domain was defined as amino acids 38–520 of the RefSeq sequence WP_014636499.1 of *S*. *suis* 05ZYH33, thereby excluding the N-terminal signal peptide and the C-terminal region only present in sequences from *S*. *sui*s. Only sequences that, when used as query in a second round of homology searches, had an overlap with the region matched in the first round and carried the conserved catalytic cysteine residue were kept for further analysis (Table 3).

**Table 3:** Number of identified putative IgdE family protease sequences and searched genomes.

*Streptococcus* species	identified putative IgdE family protease sequences	searched genomes	percentage of genomes encoding putative IgdE family protease sequences [%]
***S*. *agalactiae***	171	309	55
***S*. *dysgalactiae***	13	13	100
***S*. *equi***	6	13	46
***S*. *suis***	79	98	81
***S*. *porcinus***	1	1	100
***S*. *pseudoporcinus***	2	2	100
***S*. *canis***	1	1	100
***S*. *castoreus***	1	1	100
***S*. *merionis***	2	1	100

After homology searches and filtering steps, 55 unique sequences were identified representing putative IgdE family proteases, 23 from *S*. *suis*, 18 from S. *agalactiae*, five from *S*. *dysgalactiae*, three from *S*. *equi* subsp. *zooepidemicus* and one each from *S*. *porcinus*, *S*. *pseudoporcinus*, *S*. *canis* and *S*. *castoreus* respectively. Two sequences from the same genome of *S*. *merionis* were identified. All sequences from *S*. *agalactiae* are very similar in the defined IgdE_domain region, often only differing in one or a few amino acid positions.

**Fig 1:**
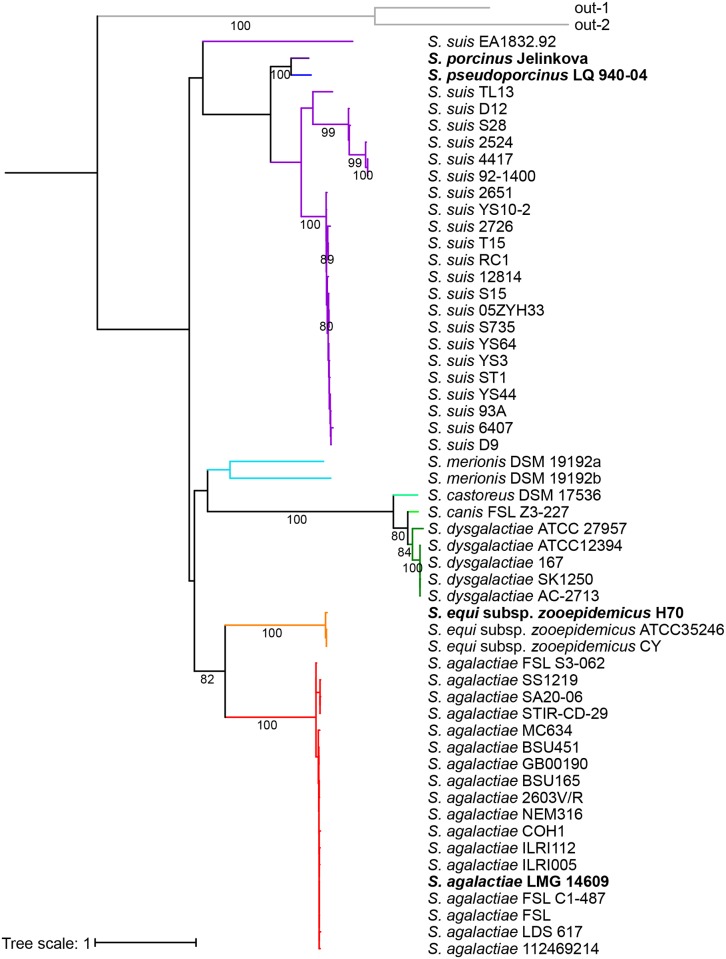
Phylogenetic tree of identified putative IgdE proteases. Phylogenic analysis of the IgdE_domain of 55 identified putative IgdE proteases. The maximum likelihood (ML) tree was constructed using a Jones-Taylor-Thornton (JTT) model with PhyML. Bootstrap values greater than or equal to 80% are shown. Putative IgdE protease sequences that corresponded to the genes cloned for expression of recombinant protein are marked in **bold**. Tree scale is given as average number of substitutions per site.

To illustrate the relationship of these 55 sequences a phylogenetic tree was inferred by maximum likelihood based on the JTT model of sequence evolution (Fig 1). The IgdE_domain region sequences of *S*. *canis* and *S*. *castoreus* showed similarity to the IgdE_domain region sequences of *S*. *dysgalactiae*. The two IgdE_domain sequences of *S*. *merionis* grouped together, but were still distinct from each other. IgdE_domain region sequences of all *S*. *agalactiae* strains grouped close together, as did sequences of *S*. *equi* and *S*. *dysgalactiae*, while the sequences of *S*. *suis* were more diverse. Also the two sequences obtained from *S*. *porcinus* and *S*. *pseudoporcinus* grouped close together.

### Locations of *igdE* genes within *Streptococcus* genomes

Each gene encoding a putative IgdE protease was localized in the respective genome as well as genes flanking the *igdE* gene (Table 4). Location of the *igdE* genes was generally conserved within species, although two out of 18 *S*. *agalactiae* strains and one out of 23 *S*. *suis* strains had deviant neighboring genes. Location and neighboring genes were conserved between the species *S*. *castoreus*, *S*. *canis* and *S*. *dysgalactiae*. For sequences retrieved from genome drafts the locations could not be determined or could only be approximated.

**Table 4:** Location of *igdE* genes within the genomes of the respective *Streptococcus* species and flanking genes.

Species	Flanking genes	Location (nucleotide number)
*S*. *agalactiae* (16)	Between "4-Hydroxy-2-oxoglutarate aldolase (EC 4.1.3.16)" and "Nitroreductase family protein"	~ 1790000–1890000
*S*. *agalactiae* (1)	Between "Voltage-gated chloride channel family protein" and "Transcriptional regulator, MarR family"	?
*S*. *agalactiae* (1)	Between "4-Hydroxy-2-oxoglutarate aldolase (EC 4.1.3.16)" and "sensor histidine kinase"	?
*S*. *castoreus* (1)	Between "UDP-N-acetylmuramoylalanine—D-glutamate ligase" and "GTP-binding protein TypA/BipA"	~ 1440000–1540000
*S*. *canis* (1)		
*S*. *dysgalactiae* (5)		
*S*. *equi* subsp. *zooepidemicus* (3)	Between "S-adenosylmethionine:tRNA ribosyltransferase-isomerase" and "Manganese superoxide dismutase "	~657000
		~1210000
		~1500000
*S*. *merionis* (1)	Between "SatD" and "tRNA:m(5)U-54 MTase gid"	?
*S*. *merionis* (1)	Between "Transmembrane component MtsC of energizing module of methionine-regulated ECF transporter" and "putative toxic anion resistance protein"	?
*S*. *porcinus* (1)	Between "Glutathione S-transferase, omega" and "Two-component system response regulator"	?
*S*. *pseudoporcinus* (1)		
*S*. *suis* (22)	Between "Protein export cytoplasm protein SecA ATPase RNA helicase" and "Fructokinase"	~ 1600000–1860000
*S*. *suis* (1)	Between "Choline binding protein A" and "Uridine kinase"	?

### IgdE family proteases are highly specific for IgG of different host species

The *igdE* genes from one representative strain of *S*. *agalactiae*, *S*. *porcinus*, *S*. *pseudoporcinus* and *S*. *equi* subsp. *zooepidemicus* (corresponding to the sequences marked in **bold** in Fig 1) were cloned into expression vectors in *E*. *coli*. The encoded proteins were over-expressed and recombinant protein was used for substrate screening by overnight incubation with potential substrates prior to analyses by SDS-PAGE and western blots.

**Fig 2:**
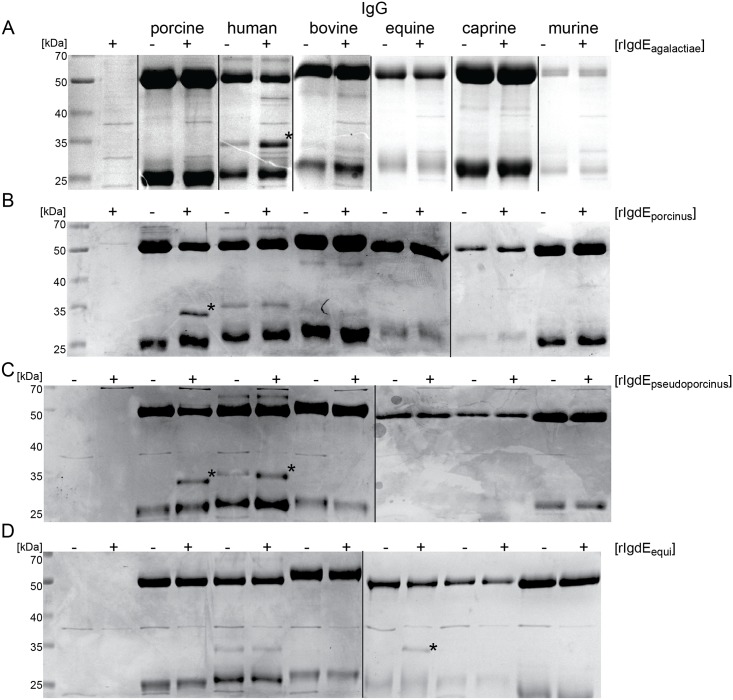
IgG host species specificity of IgdE family proteases. 0.5 mg/ml human, porcine, equine, bovine and murine IgG were incubated for 16h at 37°C with (A) 20 μg/ml purified rIgdE_agalactiae_, (B) 20 μg/ml purified rIgdE_porcinus_, (C) 5% soluble fraction of *E*. *coli* cells expressing rIgdE_pseudoporcinu_s, (D) 5% soluble fraction of *E*. *coli* cells expressing rIgdE_equi_. PBS (A and B) or 5% soluble fraction of E. coli cells without recombinant construct (C and D) were used as negative controls (-). Reactions were analyzed by Coomassie blue SDS-PAGE under reducing conditions. Images of different SDS-PAGE run in parallel have been assembled into one figure. The diagnostic 32 kDa IgG cleavage product (*) appeared when rIgdE_agalactiae_ was incubated with human IgG, rIgdE_porcinus_ with porcine IgG, rIgdE_pseudoporcinus_ with human IgG and porcine IgG, and rIgdE_equi_ with equine IgG.

Recombinant IgdE_agalactiae_ was able to degrade human IgG and a diagnostic cleavage product of 32 kDa appeared, when rIgdE_agalactiae_ was incubated with human IgG, but not when incubated with porcine, bovine, equine, caprine or murine IgG (Fig 2A). Similar to that rIgdE_porcinus_ was only able to degrade porcine IgG; the diagnostic cleavage product of 32 kDa appeared only when incubated with porcine IgG, but not when incubated with human, bovine, equine, caprine or murine IgG (Fig 2B). Recombinant IgdE_pseudoporcinus_ had in contrast dual substrate specificity towards human and porcine IgG, again characterized through appearance of diagnostic cleavage products of 32 kDa, while no degradation of bovine, equine, caprine or murine IgG could be observed (Fig 2C). Recombinant IgdE_equi_ possessed degrading activity against equine IgG, while no degradation of human, porcine, bovine, caprine or murine IgG could be observed (Fig 2D).

**Fig 3:**
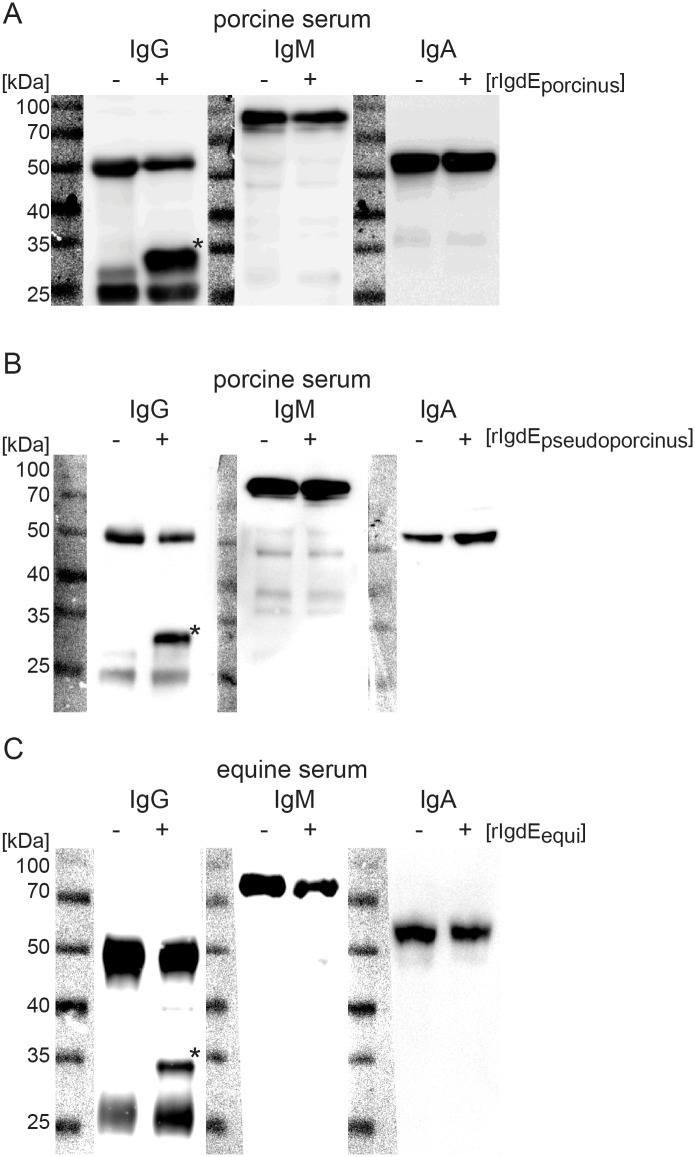
IgdE family proteases are specific for IgG compared to IgM and IgA. 2% porcine plasma was incubated with (+) or without (-) 20 μg/ml purified rIgdE_porcinus_ (A) or 5% soluble fraction of E. coli cells expressing rIgdE_pseudoporcinus_ (B) respectively for 16h at 37°C. 2% equine serum was incubated with 5% soluble fraction of E. coli cells expressing rIgdE_equi_ (C) for 16h at 37°C. The reactions were analyzed by anti-porcine or anti-equine IgG, IgM and IgA Western blots under reducing conditions. Only degradation products of IgG (*) could be observed.

IgG specificity of these novel members of the IgdE protease family was investigated by incubation of recombinant protein with porcine and equine serum, respectively. These cleavage reactions were analyzed by anti-IgG, anti-IgM and anti-IgA western blots. Recombinant IgdE_porcinus_ degraded porcine serum IgG, but not IgM or IgA (Fig 3A). A similar observation was made when serum was incubated with rIgdE_pseudoporcinus_ (Fig 3B), which also was also specific for IgG. Recombinant IgdE_equi_ cleaved equine serum IgG, but not IgM or IgA (Fig 3C).

**Fig 4:**
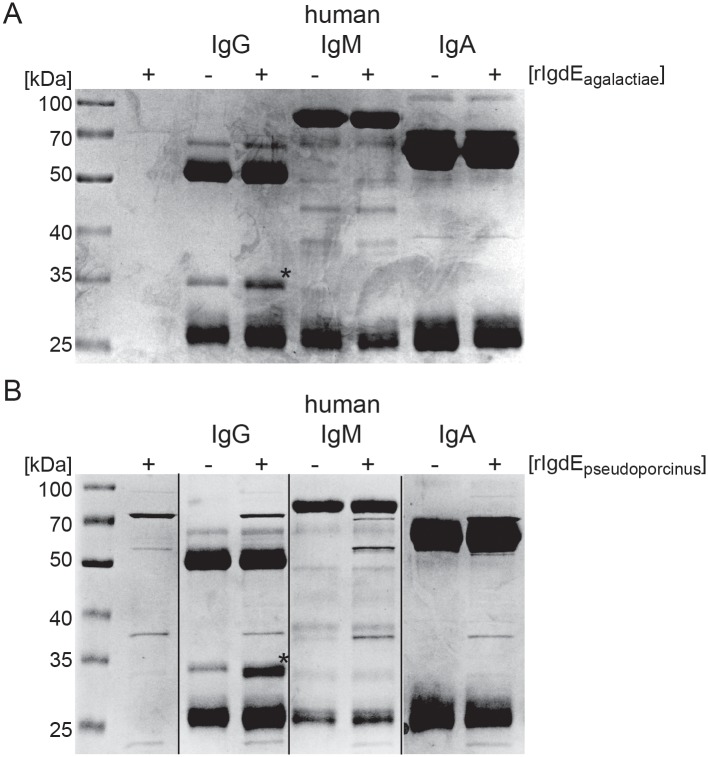
IgdE_agalactiae_ and IgdE_pseudoporcinus_ are specific for human IgG compared to IgM and IgA. 0.5 mg/ml human IgG, IgM and IgA were incubated for 16h at 37°C with (+) or without (-) 20 μg/ml purified rIgdE_agalactiae_ (A) or 5% soluble fraction of E. coli cells expressing rIgdE_pseudoporcinus_ (B). Reactions were analyzed by SDS-PAGE under reducing conditions. SDS-PAGE was stained with Coomassie blue. Order of lanes within SDS-PAGE was adjusted.

Specificity of the IgdE proteases from *S*. *agalactiae* and *S*. *pseudoporcinus* for human IgG in comparison to human IgM and IgA was tested through incubation of recombinant protein with purified Ig (all Sigma) and analyzed by reducing SDS-PAGE. The findings showed that rIgdE_agalactiae_ (Fig 4A) and rIgdE_pseudoporcinus_ (Fig 4B) were specific for IgG and cleaved human IgG, but not human IgM or IgA.

### IgG subtype specificity of IgdE proteases of *S*. *agalactiae*, *S*. *pseudoporcinus* and *S*. *equi* subsp. *zooepidemicus*

**Fig 5:**
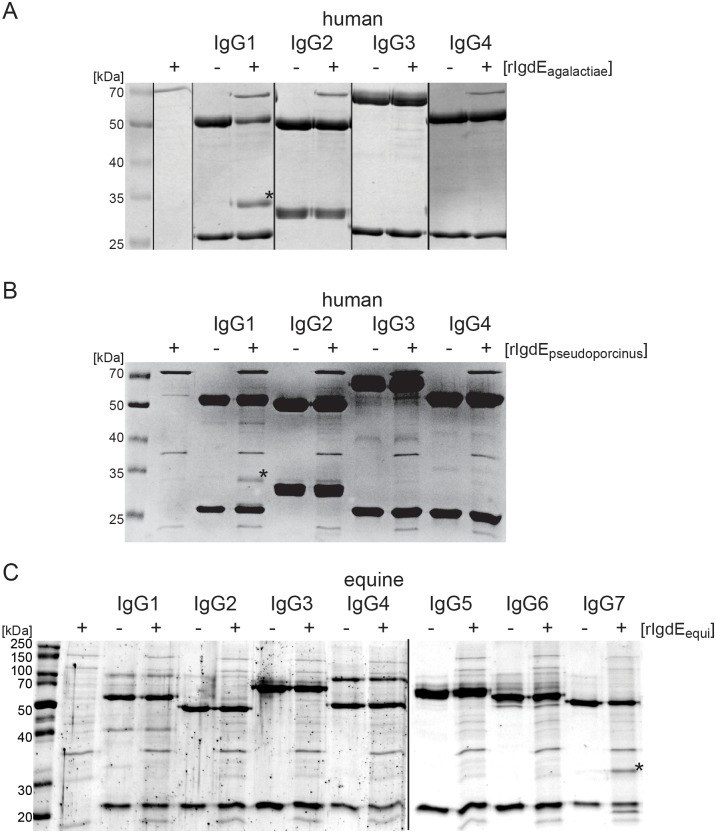
IgdE_agalactiae_, IgdE_pseudoporcinus_ and IgdE_equi_ are IgG subtype specific. (A) 0.25 mg/ml human IgG subtypes were incubated for 16h at 37°C with (+) or without (-) 20 μg/ml purified rIgdE_agalactiae_. Reactions were analyzed by SDS-PAGE under reducing conditions. IgG cleavage (*) occurred only upon incubation with IgG1. SDS-PAGE was stained with Coomassie blue. Order of lanes within SDS-PAGE was adjusted. (B) 0.25 mg/ml human IgG subtypes were incubated for 16h at 37°C with (+) or without (-) 5% soluble fraction of *E*. *coli* cells expressing rIgdE_pseudoporcinus_. Reactions were analyzed by SDS-PAGE under reducing conditions. IgG cleavage (*) occurred only upon incubation with IgG1. SDS-PAGE was stained with Coomassie blue. (C) 0.09 mg/ml recombinant equine IgG subtypes were incubated for 16h at 37°C with (+) or without (-) 5% soluble fraction of E. coli cells expressing rIgdE_equi_. Reactions were analyzed by SDS-PAGE under reducing conditions. IgG cleavage (*) occurred only upon incubation with IgG7. SDS-PAGE was stained with Coomassie Fluor Orange Protein Gel Stain. Images of different SDS-PAGE run in parallel have been assembled into one figure.

Given the shown specificity of IgdE proteases for IgG of specific hosts, we were interested in IgG subtype specificity of these proteases. Recombinant IgdE_agalactiae_ (Fig 5A) and rIgdE_pseudoporcinus_ (Fig 5B) were therefore incubated with human IgG1, IgG2, IgG3 and IgG4 from myeloma source prior to reducing SDS-PAGE analysis. Both rIgdE_agalactiae_ and rIgdE_pseudoporcinus_ were strictly IgG1 specific, and no degradation of human IgG2, IgG3 and IgG4 was observed. The equine IgG subtype specificity of rIgdE_equi_ was tested by incubation of protease preparations with purified recombinant equine IgG1, IgG2, IgG3, IgG4, IgG5, IgG6 and IgG7 (Fig 5C). Strikingly, pronounced subtype specificity was also observed for rIgdE_equi_ and of all equine IgG subtypes only recombinant equine IgG7 was cleaved.

### Cleavage sites of IgdE family proteases

**Fig 6:**
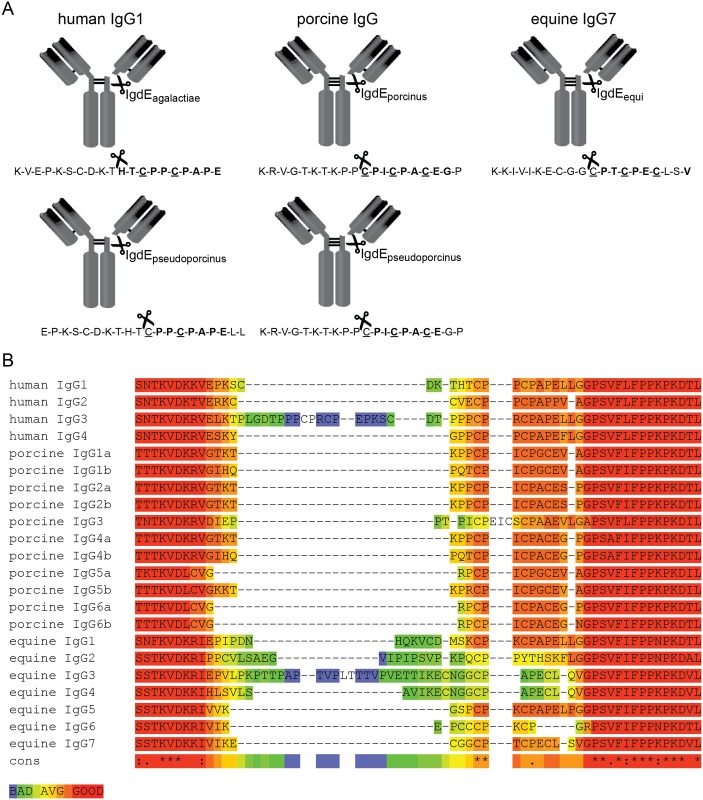
IgdE family proteases cleave IgG in the hinge region. (A) The cleavage sites within IgG molecules were determined through N-terminal Edman sequencing of the 32 kDa IgG cleavage products generated by IgdE family proteases. The identified aa sequences (**bold**) were found in the hinge regions of the respective IgG heavy chains. Homodimer disulfide bond cysteine residues are underlined. 10 aa N- and C-terminal from the identified cleavage site (scissor symbol) of the respective IgG heavy chain are shown. Porcine IgG4a was chosen as a representative for porcine IgG. (B) Sequences of the hinge regions and adjacent parts of the CH1 and CH2 domains of human, porcine and equine IgG subtypes were aligned using T-COFFEE (Version_8.93) [38] to illustrate hinge region diversity. Alignment reliability assessed by TCS [39] is color coded (blue to red).

To determine the exact cleavage site of the IgdE proteases within the respective substrate IgG molecules, the 32 kDa cleavage products generated by IgdE_agalactiae_, IgdE_porcinus_, IgdE_pseudoporcinus_, and IgdE_equi_ were subjected to N-terminal Edman sequencing. All obtained sequences corresponded to the hinge region of the respective IgG molecules (Fig 6A). IgdE_agalactiae_ and IgdE_pseudoporcinus_ cleave both human IgG1, but cleavage sites are not identical, being located two residues apart from each other. IgdE_agalactiae_ cleaves the human IgG1 heavy chain two residues N-terminal of the putative homodimer disulfide bond cysteine residues; while all other cleavage sites of IgdE family proteases are located directly adjacent to the putative homodimer disulfide bond cysteine residues in IgG hinge regions. The cleavage sites identified in porcine IgG generated through cleavage with both IgdE_porcinus_ and IgdE_pseudoporcinus_ were identical and found in porcine IgG2, IgG4 and IgG6 (Fig 6B). Similar sequences can, however, also be found in IgG1 and IgG5. The sequence obtained by N-terminal Edman sequencing of the cleavage product of equine IgG generated by IgdE_equi_ corresponded to the hinge region of equine IgG7. The amino acid sequences analogous to those adjacent to the cleavage site in equine IgG7 are different in all the other horse IgG subtypes, providing a rationale for the observed subtype specificity.

## Discussion

Based on the identification of the founding member of a novel cysteine protease family, IgdE of *S*. *suis* [2], we identified several putative IgdE family proteases through homology searches within the genus *Streptococcus*. Locations of the genes of these putative proteases were conserved within, and to certain degree also between, different *Streptococcus* species suggesting that *igdE* genes are part of the core genome and not part of mobile elements (Table 4). One sequence of *S*. *suis* and two of *S*. *agalactiae* had, however, different neighboring genes than the other 22 sequences of *S*. *suis* and 16 of *S*. *agalactiae*, respectively. Putative IgdE family protease sequences were however only found in 55% of *S*. *agalactiae*, 46% of *S*. *equi* and 81% of *S*. *suis* genomes (Table 3). This could be due to the real absence of an *igdE* gene in some strains of these species or due to pore, incomplete or missanotated coding sequences of these genomes.

All investigated IgdE family proteases except IgdE_pseudoporcinus_, showed only specificity towards IgG of one host species. The substrate specificity of these IgdE family proteases correlates well with the known host tropism of the respective *Streptococcus* species. However, IgdE_agalactiae_ does not cleave bovine IgG, despite *S*. *agalactiae* being the cause of mastitis in cattle besides being an important human pathogen. The observed substrate preference for human IgG1 might reflect that most human invasive *S*. *agalactiae* isolates represent distinct subtypes from bovine isolates, as it has been suggested in a temporally and geographically matched isolate characterization study [40]. IgdE_pseudoporcinus_ showed double specificity for both human IgG1 and porcine IgG (Fig 2). Since *S*. *pseudoporcinus* is closely related to the pig pathogen *S*. *porcinus*, the specificity for porcine IgG is not that surprising. It is, however, astonishing that IgdE_pseudoporcinus_, being a human pathogen, has evolved the ability to cleave human IgG1, despite the close relationship to IgdE_porcinus_ that does not possess this ability. Thus, it seems advantageous for *S*. *pseudoporcinus* to have a human IgG1 degrading protease, underlining the importance of IgG1 in immune responses towards bacterial pathogens. Mutational studies of IgdE_pseudoporcinus_ and IgdE_porcinus_ might be able to dissect the residues that mediate this substrate specificity.

We suggest that substrate specificity of IgdE family proteases contribute to the host tropism of some *Streptococcus* species. Co-evolution of streptococcal opportunistic pathogens and their host could be reflected on a molecular level by co-evolution of IgdE family proteases and their substrate IgG heavy chain molecules. This is highly reminiscent of the co-evolution described between IgA and bacterial proteins targeting IgA, such as IgA-binding proteins and IgA-specific proteases, where reiterative episodes of natural selection are predicted to have shaped the interactions between the IgA and the bacterial proteins, reflecting an ‘arms race’ [41, 42].

The subtype specificity of the IgdE family proteases of *S*. *agalactiae* and *S*. *pseudoporcinus* towards human IgG1 and the IgdE family protease of *S*. *equi* subsp. *zooepidemicus* towards equine IgG7 is striking and surprising (Fig 5). The evolutionary benefit for streptococci to possess such IgG subtype specific proteases compared to proteases with broader specificity is at the first glance puzzling. Cleavage of these IgG subtypes might, however, be sufficient to overcome key immune defense mechanisms in certain niches. For example, *S*. *agalactiae* is a common cause of invasive neonatal infections in humans [43] and human IgG1 is, along with IgG3, the major human Ig transported across the placental barrier [44]. Thereby cleavage of human IgG1 might be sufficient to evade the Ig mediated immune response in the newborn human host. Moreover, targeted disruption of IgG7 function by *S*. *equi* in the horse is likely to significantly comprise IgG-mediated protection, given that IgG7 is one of the predominant subclasses in equine serum [45]. Due to the high diversity in the hinge region of different IgG subtypes [17, 46] it might also be difficult to evolve IgG degrading proteases with broader specificity. Since some IgG subtypes, for example human IgG4, are believed to mediate tolerance [47, 48] it might even be beneficial for an opportunistic pathogen to carry proteases incapable of cleaving these IgG subtypes. Investigations on the potential role of IgdE_agalactiae_ in immune evasion in the neonatal and adult host are currently ongoing in our group.

IgdE_equi_ is the third IgG degrading protease of *S*. *equi* subsp. *zooepidemicus* described, beside IdeZ1 [5] and IdeZ2 [49]. IgdE_equi_ is highly specific for equine IgG7, while both IdeZ1 and IdeZ2 have broader specificity, cleaving IgG of several host species. The abundance of genes encoding IgG degrading proteases in *S*. *equi* subsp. *zooepidemicus* implicates a special importance of an IgG cleaving phenotype of this species. These Ig-degrading proteases might be regulated by different gene regulation systems and thereby expressed during different stages of infection or colonization.

All IgdE family proteases recognize IgG as substrates although the amino acid sequences at the cleavage sites in the respective hinge regions are quite diverse (Fig 6). Therefore preference for IgG as substrates of IgdE family proteases might not only be conferred by the actual cleavage site, but also by motifs lying adjacent to it or within the Fc fragment or F(ab) fragment. Indeed, this possibility mirrors that observed with certain human IgA1 proteases in that residues within the Fc region of IgA have been shown to be essential for recognition of human IgA1 as a substrate for cleavage [50, 51]. Further parallels with IgA1 protease cleavage of IgA1 hinge and the cleavage of IgG hinges by IgdE family proteases described here can be noted. Different IgA1 proteases are known to cleave at different specific peptide bonds in the IgA1 hinge sequence, and evidence suggests that for cleavage to occur each protease has a requirement for the Fab and Fc regions to be separated by a particular number of amino acids, presumably to allow appropriate access and orientation of the protease [52]. Possibly similar spatial considerations may impact on the ability of IgdE family members to cleave their respective substrates, and may provide a further explanation for their exquisite specificity for particular IgG subtypes.

IgdE_agalactiae_ has the same cleavage site in human IgG1 as papain [53]. However compared to papain, IgdE_agalactiae_ is highly specific for human IgG1 and has only one distinct cleavage site within the heavy chain. Interestingly, this cleavage site is not shared by IgdE_pseudoporcinus_ that instead cleaves the heavy chain two residues closer to the C-terminus, just N-terminal of the putative homodimer disulfide bond cysteine residue. Differential cleavage sites implicate that targeting IgG1 has evolved independently in these two proteases, highlighting the importance for the bacteria to counteract IgG1. The cleavage sites of IgdE_porcinus_, IgdE_pseudoporcinus_ and IgdE_equi_ have a CPxCP motif just C-terminal of the cleavage site in common. This motif can, however, also be found in many IgG heavy chain molecules that are not substrates of any investigated IgdE family protease, supporting the idea that substrate specificity is determined by features others than cleavage site sequences.

Secreted Ig degrading proteases have been shown to be protective antigens in experimental vaccine and infection studies with *S*. *suis* in pigs [54] and *S*. *equi* in horses [55]. The described IgdE proteases might therefore be suitable vaccine targets. Given the homology of these proteases, especially in regions close to the active site, vaccination might even give cross protection against several *Streptococcus* species. This would especially be desirable in the cases of *S*. *agalactiae* and *S*. *pseudoporcinus* in humans and *S*. *suis* and *S*. *porcinus* in pigs. Antibodies elicited by such vaccines might both neutralize the proteolytic function of these potential immune evasion factors and potentially mediate antibody dependent cell cytotoxicity against the streptococcal pathogen.

Lastly IgdE family proteases with pronounced species and subtype specificity might also be of biotechnological or therapeutical use, *i*.*e*. similar to what has been proven for the IgG degrading enzyme of *S*. *pyogenes* IdeS [56–61].

## References

[1] Facklam R. What Happened to the Streptococci: Overview of Taxonomic and Nomenclature Changes. Clin Microbiol Rev. 152002. p. 613–30. 10.1128/CMR.15.4.613-630.2002 12364372 PMC126867

[2] Spoerry C, Seele J, Valentin-Weigand P, Baums CG, von Pawel-Rammingen U. Identification and Characterization of IgdE, a Novel IgG-degrading Protease *of Streptococcus suis* with Unique Specificity for Porcine IgG. J Biol Chem. 2016;291(15):7915–25. 10.1074/jbc.M115.711440 26861873 PMC4824999

[3] Rawlings ND, Waller M, Barrett AJ, Bateman A. MEROPS: the database of proteolytic enzymes, their substrates and inhibitors. Nucleic Acids Res. 2014;42(Database issue):D503–9. Epub 2013/10/26. 10.1093/nar/gkt953 24157837 PMC3964991

[4] von Pawel-Rammingen U, Johansson BP, Bjorck L. IdeS, a novel streptococcal cysteine proteinase with unique specificity for immunoglobulin G. Embo j. 2002;21(7):1607–15. Epub 2002/04/03. 10.1093/emboj/21.7.1607 11927545 PMC125946

[5] Lannergard J, Guss B. IdeE, an IgG-endopeptidase of *Streptococcus equi* ssp. *equi*. FEMS Microbiol Lett. 2006;262(2):230–5. Epub 2006/08/23. 10.1111/j.1574-6968.2006.00404.x . 16923080

[6] Kilian M, Mestecky J, Schrohenloher RE. Pathogenic species of the genus *Haemophilus* and *Streptococcus pneumoniae* produce immunoglobulin A1 protease. Infect Immun. 1979;26(1):143–9. Epub 1979/10/01. 40878; PubMed Central PMCID: PMCPMC414586. 40878 10.1128/iai.26.1.143-149.1979 PMC414586

[7] Reinholdt J, Tomana M, Mortensen SB, Kilian M. Molecular aspects of immunoglobulin A1 degradation by oral streptococci. Infect Immun. 1990;58(5):1186–94. 2182537 10.1128/iai.58.5.1186-1194.1990 PMC258608

[8] Seele J, Singpiel A, Spoerry C, von Pawel-Rammingen U, Valentin-Weigand P, Baums CG. Identification of a novel host-specific IgM protease in *Streptococcus suis*. J Bacteriol. 195 United States2013 p. 930–40. 10.1128/JB.01875-12 23243300 PMC3571317

[9] Markowska-Daniel I, Pomorska-Mól M, Pejsak Z. Dynamic changes of immunoglobulin concentrations in pig colostrum and serum around parturition. Pol J Vet Sci. 2010;13(1):21–7. . 21077427

[10] Panda S, Ding JL. Natural antibodies bridge innate and adaptive immunity. J Immunol. 2015;194(1):13–20. 10.4049/jimmunol.1400844 . 25527792

[11] Schur PH. IgG subclasses. A historical perspective. Monogr Allergy. 1988;23:1–11. . 3290655

[12] Simister NE. Placental transport of immunoglobulin G. Vaccine. 2003;21(24):3365–9. Epub 2003/07/10. . 12850341 10.1016/s0264-410x(03)00334-7

[13] Bruhns P, Iannascoli B, England P, Mancardi DA, Fernandez N, Jorieux S, et al. Specificity and affinity of human Fcgamma receptors and their polymorphic variants for human IgG subclasses. Blood. 2009;113(16):3716–25. 10.1182/blood-2008-09-179754 . 19018092

[14] Bindon CI, Hale G, Brüggemann M, Waldmann H. Human monoclonal IgG isotypes differ in complement activating function at the level of C4 as well as C1q. J Exp Med. 1988;168(1):127–42. 3260935 10.1084/jem.168.1.127 PMC2188986

[15] Schumaker VN, Calcott MA, Spiegelberg HL, Müller-Eberhard HJ. Ultracentifuge studies of the binding of IgG of different subclasses to the Clq subunit of the first component of complement. Biochemistry. 1976;15(23):5175–81. . 990273 10.1021/bi00668a035

[16] Tao MH, Smith RI, Morrison SL. Structural features of human immunoglobulin G that determine isotype-specific differences in complement activation. J Exp Med. 1993;178(2):661–7. 8340761 10.1084/jem.178.2.661 PMC2191116

[17] Vidarsson G, Dekkers G, Rispens T. IgG subclasses and allotypes: from structure to effector functions. Front Immunol. 2014;5:520 10.3389/fimmu.2014.00520 25368619 PMC4202688

[18] Butler JE, Wertz N. Antibody repertoire development in fetal and neonatal piglets. XVII. IgG subclass transcription revisited with emphasis on new IgG3. J Immunol. 2006;177(8):5480–9. . 17015734 10.4049/jimmunol.177.8.5480

[19] Butler JE, Wertz N, Deschacht N, Kacskovics I. Porcine IgG: structure, genetics, and evolution. Immunogenetics. 2009;61(3):209–30. Epub 2008/12/03. 10.1007/s00251-008-0336-9 . 19048248

[20] Wagner B, Miller DC, Lear TL, Antczak DF. The complete map of the Ig heavy chain constant gene region reveals evidence for seven IgG isotypes and for IgD in the horse. J Immunol. 2004;173(5):3230–42. . 15322185 10.4049/jimmunol.173.5.3230

[21] Lewis MJ, Wagner B, Woof JM. The different effector function capabilities of the seven equine IgG subclasses have implications for vaccine strategies. Mol Immunol. 2008;45(3):818–27. Epub 2007/08/03. 10.1016/j.molimm.2007.06.158 17669496 PMC2075531

[22] Bliss SJ, Manning SD, Tallman P, Baker CJ, Pearlman MD, Marrs CF, et al. Group B *Streptococcus* colonization in male and nonpregnant female university students: a cross-sectional prevalence study. Clin Infect Dis. 2002;34(2):184–90. Epub 2001/12/12. 10.1086/338258 . 11740706

[23] Ippolito DL, James WA, Tinnemore D, Huang RR, Dehart MJ, Williams J, et al. Group B *streptococcus* serotype prevalence in reproductive-age women at a tertiary care military medical center relative to global serotype distribution. BMC Infect Dis. 2010;10:336 10.1186/1471-2334-10-336 21106080 PMC3004907

[24] Yang Y, Liu Y, Ding Y, Yi L, Ma Z, Fan H, et al. Molecular characterization of *Streptococcus agalactiae* isolated from bovine mastitis in Eastern China. PLoS One. 2013;8(7):e67755 10.1371/journal.pone.0067755 23874442 PMC3707890

[25] Sunkara B, Bheemreddy S, Lorber B, Lephart PR, Hayakawa K, Sobel JD, et al. Group B *Streptococcus* infections in non-pregnant adults: the role of immunosuppression. Int J Infect Dis. 2012;16(3):e182–6. 10.1016/j.ijid.2011.11.008 . 22236484

[26] O'Sullivan T, Friendship R, Blackwell T, Pearl D, McEwen B, Carman S, et al. Microbiological identification and analysis of swine tonsils collected from carcasses at slaughter. Can J Vet Res. 2011;75(2):106–11. 21731180 PMC3062919

[27] Hajtos I, Glavits R, Makrai L, Hallgatone IS, Jochman J. Occurrence of porcine purulent lymphadenitis caused by *Streptococcus porcinus* in Hungary. Magyar Allatorvosok Lapja. 2002;124(3):161–8. WOS:000174545500005.

[28] Lammler C, Bahr KH. Characterisation of *Streptococcus porcinus* serogroup P isolated from an aborted fetus of a pig. Medical Science Research. 1996;24(3):177–8. WOS:A1996UF45700014.

[29] Bekal S, Gaudreau C, Laurence RA, Simoneau E, Raynal L. *Streptococcus pseudoporcinus* sp *nov*., a novel species isolated from the genitourinary tract of women. Journal of Clinical Microbiology. 2006;44(7):2584–6. 10.1128/jcm.02707-05. WOS:000239157400045. 16825387 PMC1489492

[30] Stoner KA, Rabe LK, Austin MN, Meyn LA, Hillier SL. Incidence and epidemiology of *Streptococcus pseudoporcinus* in the genital tract. J Clin Microbiol. 2011;49(3):883–6. Epub 2010/12/31. 10.1128/jcm.01965-10 21191057 PMC3067687

[31] Pelkonen S, Lindahl SB, Suomala P, Karhukorpi J, Vuorinen S, Koivula I, et al. Transmission of *Streptococcus equi* subspecies *zooepidemicus* infection from horses to humans. Emerg Infect Dis. 2013;19(7):1041–8. 10.3201/121365 23777752 PMC3713971

[32] Timoney JF. The pathogenic equine streptococci. Vet Res. 2004;35(4):397–409. 10.1051/vetres:2004025 15236673

[33] Jorm LR, Love DN, Bailey GD, McKay GM, Briscoe DA. Genetic structure of populations of beta-haemolytic Lancefield group C streptococci from horses and their association with disease. Res Vet Sci. 1994;57(3):292–9. Epub 1994/11/01. . 7871247 10.1016/0034-5288(94)90120-1

[34] Sievers F, Wilm A, Dineen D, Gibson TJ, Karplus K, Li W, et al. Fast, scalable generation of high-quality protein multiple sequence alignments using Clustal Omega. Molecular Systems Biology. 2011;7(1). 10.1038/msb.2011.75 21988835 PMC3261699

[35] Darriba D, Taboada GL, Doallo R, Posada D. ProtTest 3: fast selection of best-fit models of protein evolution. Bioinformatics. 2011;27(8):1164–5. Epub 2011/02/22. 10.1093/bioinformatics/btr088 . 21335321 PMC5215816

[36] Guindon S, Gascuel O. A simple, fast, and accurate algorithm to estimate large phylogenies by maximum likelihood. Syst Biol. 2003;52(5):696–704. Epub 2003/10/08. . 14530136 10.1080/10635150390235520

[37] Letunic I, Bork P. Interactive Tree Of Life (iTOL): an online tool for phylogenetic tree display and annotation. Bioinformatics. 2007;23(1):127–8. Epub 2006/10/20. 10.1093/bioinformatics/btl529 . 17050570

[38] Notredame C, Higgins DG, Heringa J. T-Coffee: A novel method for fast and accurate multiple sequence alignment. J Mol Biol. 2000;302(1):205–17. 10.1006/jmbi.2000.4042 . 10964570

[39] Chang JM, Di Tommaso P, Notredame C. TCS: a new multiple sequence alignment reliability measure to estimate alignment accuracy and improve phylogenetic tree reconstruction. Mol Biol Evol. 2014;31(6):1625–37. 10.1093/molbev/msu117 . 24694831

[40] Sukhnanand S, Dogan B, Ayodele MO, Zadoks RN, Craver MP, Dumas NB, et al. Molecular subtyping and characterization of bovine and human *Streptococcus agalactiae* isolates. J Clin Microbiol. 2005;43(3):1177–86. 10.1128/JCM.43.3.1177-1186.2005 15750080 PMC1081236

[41] Abi-Rached L, Dorighi K, Norman PJ, Yawata M, Parham P. Episodes of natural selection shaped the interactions of IgA-Fc with FcalphaRI and bacterial decoy proteins. J Immunol. 2007;178(12):7943–54. . 17548632 10.4049/jimmunol.178.12.7943

[42] Pinheiro A, Woof JM, Abi-Rached L, Parham P, Esteves PJ. Computational analyses of an evolutionary arms race between mammalian immunity mediated by immunoglobulin A and its subversion by bacterial pathogens. PLoS One. 2013;8(9):e73934 10.1371/journal.pone.0073934 24019941 PMC3760800

[43] Bekker V, Bijlsma MW, van de Beek D, Kuijpers TW, van der Ende A. Incidence of invasive group B streptococcal disease and pathogen genotype distribution in newborn babies in the Netherlands over 25 years: a nationwide surveillance study. Lancet Infect Dis. 2014;14(11):1083–9. Epub 2014/12/03. 10.1016/s1473-3099(14)70919-3 . 25444407

[44] Simister NE, Story CM, Chen HL, Hunt JS. An IgG-transporting Fc receptor expressed in the syncytiotrophoblast of human placenta. Eur J Immunol. 1996;26(7):1527–31. 10.1002/eji.1830260718 . 8766556

[45] Sheoran AS, Timoney JF, Holmes MA, Karzenski SS, Crisman MV. Immunoglobulin isotypes in sera and nasal mucosal secretions and their neonatal transfer and distribution in horses. Am J Vet Res. 2000;61(9):1099–105. Epub 2000/09/08. . 10976743 10.2460/ajvr.2000.61.1099

[46] Ellison JW, Berson BJ, Hood LE. The nucleotide sequence of a human immnnoglobulin Cγl gene. Nucleic Acids Research. 1982;10(13):4071–9. 10.1093/nar/10.13.4071 6287432 PMC320779

[47] Aalberse RC, Stapel SO, Schuurman J, Rispens T. Immunoglobulin G4: an odd antibody. Clin Exp Allergy. 2009;39(4):469–77. Epub 2009/02/19. 10.1111/j.1365-2222.2009.03207.x . 19222496

[48] Jutel M, Akdis CA. Immunological mechanisms of allergen-specific immunotherapy. Allergy. 2011;66(6):725–32. Epub 2011/04/07. 10.1111/j.1398-9995.2011.02589.x . 21466562

[49] Hulting G, Flock M, Frykberg L, Lannergård J, Flock JI, Guss B. Two novel IgG endopeptidases of *Streptococcus equi*. FEMS Microbiol Lett. 2009;298(1):44–50. 10.1111/j.1574-6968.2009.01698.x . 19659725

[50] Chintalacharuvu KR, Chuang PD, Dragoman A, Fernandez CZ, Qiu J, Plaut AG, et al. Cleavage of the human immunoglobulin A1 (IgA1) hinge region by IgA1 proteases requires structures in the Fc region of IgA. Infect Immun. 2003;71(5):2563–70. 10.1128/IAI.71.5.2563-2570.2003 12704129 PMC153282

[51] Senior BW, Woof JM. Sites in the CH3 domain of human IgA1 that influence sensitivity to bacterial IgA1 proteases. J Immunol. 2006;177(6):3913–9. . 16951354 10.4049/jimmunol.177.6.3913

[52] Senior BW, Woof JM. The influences of hinge length and composition on the susceptibility of human IgA to cleavage by diverse bacterial IgA1 proteases. J Immunol. 2005;174(12):7792–9. . 15944283 10.4049/jimmunol.174.12.7792

[53] Wang AC, Wang IY. Cleavage sites of human IgG1 immunoglobulin by papain. Immunochemistry. 1977;14(3):197–200. Epub 1977/03/01. . 863464 10.1016/0019-2791(77)90194-x

[54] Seele J, Hillermann LM, Beineke A, Seitz M, von Pawel-Rammingen U, Valentin-Weigand P, et al. The immunoglobulin M-degrading enzyme of *Streptococcus suis*, IdeSsuis, is a highly protective antigen against serotype 2. Vaccine. 2015;33(19):2207–12. 10.1016/j.vaccine.2015.03.047 . 25825330

[55] Guss B, Flock M, Frykberg L, Waller AS, Robinson C, Smith KC, et al. Getting to grips with strangles: an effective multi-component recombinant vaccine for the protection of horses from *Streptococcus equi* infection. PLoS Pathog. 2009;5(9):e1000584 10.1371/journal.ppat.1000584 19763180 PMC2736577

[56] Johansson BP, Shannon O, Björck L. IdeS: a bacterial proteolytic enzyme with therapeutic potential. PLoS One. 2008;3(2):e1692 10.1371/journal.pone.0001692 18301769 PMC2253494

[57] Winstedt L, Järnum S, Nordahl EA, Olsson A, Runström A, Bockermann R, et al. Complete Removal of Extracellular IgG Antibodies in a Randomized Dose-Escalation Phase I Study with the Bacterial Enzyme IdeS—A Novel Therapeutic Opportunity. PLoS One. 2015;10(7):e0132011 10.1371/journal.pone.0132011 26177518 PMC4503742

[58] Yang R, Otten MA, Hellmark T, Collin M, Björck L, Zhao MH, et al. Successful treatment of experimental glomerulonephritis with IdeS and EndoS, IgG-degrading streptococcal enzymes. Nephrol Dial Transplant. 2010;25(8):2479–86. 10.1093/ndt/gfq115 . 20219834

[59] Takahashi R, Yuki N. Streptococcal IdeS: therapeutic potential for Guillain-Barré syndrome. Sci Rep. 2015;5:10809 10.1038/srep10809 26194472 PMC4508529

[60] An Y, Zhang Y, Mueller HM, Shameem M, Chen X. A new tool for monoclonal antibody analysis: application of IdeS proteolysis in IgG domain-specific characterization. MAbs. 2014;6(4):879–93. 10.4161/mabs.28762 24927271 PMC4171023

[61] Nandakumar KS, Johansson BP, Björck L, Holmdahl R. Blocking of experimental arthritis by cleavage of IgG antibodies in vivo. Arthritis Rheum. 2007;56(10):3253–60. 10.1002/art.22930 . 17907170

