# Correction: Novel IgG-Degrading Enzymes of the IgdE Protease Family Link Substrate Specificity to Host Tropism of *Streptococcus* Species

**DOI:** 10.1371/journal.pone.0170880

**Published:** 2017-01-20

**Authors:** Christian Spoerry, Pontus Hessle, Melanie J. Lewis, Lois Paton, Jenny M. Woof, Ulrich von Pawel-Rammingen

Medical Research Scotland (LP) is incorrectly named in the Funding section. The correct funding information is as follows: Financial support was provided by Carl Tryggers foundation (CTS13-514) (www.carltryggersstiftelse.se/index.php?sida=rules&n=3) and Umeå University insamlingsstiftelse (2.1.12-1605-14) (www.umu.se) to UPR, Wellcome Trust (074863) to JW, with support of Kempestiftelsen (CS) and the Medical Research Scotland (LP). The funders had no role in study design, data collection and analysis, decision to publish, or preparation of the manuscript.
